# Street crossing behavior in younger and older pedestrians: an eye- and head-tracking study

**DOI:** 10.1186/s12877-015-0175-0

**Published:** 2015-12-29

**Authors:** G. A. Zito, D. Cazzoli, L. Scheffler, M. Jäger, R. M. Müri, U. P. Mosimann, T. Nyffeler, F. W. Mast, T. Nef

**Affiliations:** Gerontechnology and Rehabilitation Group, University of Bern, Bern, Switzerland; Perception and Eye Movement Laboratory, Division of Cognitive and Restorative Neurology, Department of Neurology, University Hospital Inselspital, University of Bern, Bern, Switzerland; University Hospital of Old Age Psychiatry and Psychotherapy, University of Bern, Bern, Switzerland; Center of Neurology and Neurorehabilitation, Luzerner Kantonsspital, Spitalstrasse, Luzern, Switzerland; Department of Psychology, University of Bern, Bern, Switzerland; Center for Cognition, Learning and Memory, University of Bern, Bern, Switzerland; Private Hospital Wyss, Münchenbuchsee, Switzerland; ARTORG Center for Biomedical Engineering Research, University of Bern, Bern, Switzerland

**Keywords:** Eye- and head-tracking technology, Older pedestrians, Street crossing behavior, Virtual reality

## Abstract

**Background:**

Crossing a street can be a very difficult task for older pedestrians. With increased age and potential cognitive decline, older people take the decision to cross a street primarily based on vehicles’ distance, and not on their speed. Furthermore, older pedestrians tend to overestimate their own walking speed, and could not adapt it according to the traffic conditions. Pedestrians’ behavior is often tested using virtual reality. Virtual reality presents the advantage of being safe, cost-effective, and allows using standardized test conditions.

**Methods:**

This paper describes an observational study with older and younger adults. Street crossing behavior was investigated in 18 healthy, younger and 18 older subjects by using a virtual reality setting. The aim of the study was to measure behavioral data (such as eye and head movements) and to assess how the two age groups differ in terms of number of safe street crossings, virtual crashes, and missed street crossing opportunities. Street crossing behavior, eye and head movements, in older and younger subjects, were compared with non-parametric tests.

**Results:**

The results showed that younger pedestrians behaved in a more secure manner while crossing a street, as compared to older people. The eye and head movements analysis revealed that older people looked more at the ground and less at the other side of the street to cross.

**Conclusions:**

The less secure behavior in street crossing found in older pedestrians could be explained by their reduced cognitive and visual abilities, which, in turn, resulted in difficulties in the decision-making process, especially under time pressure.

Decisions to cross a street are based on the distance of the oncoming cars, rather than their speed, for both groups. Older pedestrians look more at their feet, probably because of their need of more time to plan precise stepping movement and, in turn, pay less attention to the traffic. This might help to set up guidelines for improving senior pedestrians’ safety, in terms of speed limits, road design, and mixed physical-cognitive trainings.

## Background

Crossing a street can be a very difficult task for older pedestrians. In 2012, 17 % of all traffic fatalities in the United States involved 65 years old people and older [1]. The challenge in deciding when to safely cross a street consists of acquiring and interpreting visual and acoustic information within a limited amount of time. This poses high demands on perception (e.g., vision, hearing), and cognition (e.g., attention, processing speed) [2, 3]. Both directions of the roadway must be inspected, the vehicles and their speed detected and processed, and the pedestrians must estimate when the vehicles will arrive at the crossing point. Additionally, pedestrians have to combine the estimated arrival times of the vehicles with the estimation of their own walking speed, and judge whether the gap is long enough for a safe crossing. These visual and cognitive processes lead to the decision to either cross the road or let the vehicles pass and wait for the next opportunity. Furthermore, while crossing, the representation of the scene must be updated and, in case of wrong judgment, the walking speed must be rapidly adapted to the actual traffic situation [4].

Normal aging can affect perception and cognition, which in turn might have an impact on street crossing behavior [5]. Several studies investigated differences between the street crossing behavior of younger and older people [6–10]. These studies found that decisions of older people in selecting safe gaps were often based on simple heuristics, such as vehicle’s distance and not its speed [11, 12]. A consequence of this pattern was an imprecise time-to-arrival estimation [13]. In facts, older people often report that they think to have more time to cross a street than they actually have.

Not only visual and cognitive, but also motor abilities were shown to play an important role in street crossing behavior. In previous studies, Dommes et al. [2, 14] showed that older pedestrians overestimated their own walking speed, and could not adapt it according to the actual traffic conditions. In agreement with these results, a study by Oxley et al. [15] showed that slower walkers made more unsafe crossings than faster walkers, suggesting that slower walkers had more difficulties in adjusting their behavior to compensate for decreased motor abilities.

Since studying street crossing behavior in a real life environment may be dangerous, many studies focused on virtual reality settings [2, 11, 16, 17]. Virtual reality presents, on the one hand, the advantage of giving the participants the feeling of immersion in the virtual environment and, therefore, a realistic experience [18]. Furthermore, it is safe, cost-effective, and allows to use standardized test conditions, with a strong experimental control over the tasks [19]. On the other hand, virtual reality has certain disadvantages. The participants have to immerse themselves in the virtual scene, and it is not sure whether distances and speeds of the vehicles are perceived in the same way as in the corresponding real-life environment. Another technology often used to measure cognitive and visual abilities is eye-tracking [20]. This technique is based on the detection of some characteristics of the eye, such as the pupil shape, that vary with the eye movements and that can be detected by a camera [21]. The gaze direction is extracted by means of algorithms of image analysis, and then mapped in order to return the points on the scene where the eye looked at. Indeed, eye and head movements proved to be a valid and reliable technique to assess visual exploration behavior [22], and provide insights into the street crossing decision processes. However, to the best of our knowledge, there are no studies that explored visual exploration behavior of younger and older pedestrians during a street crossing task.

In the present study, street crossing behavior was investigated in healthy, younger and older subjects by means of a virtual reality setting. The aim of the study was to measure behavioral data (such as eye and head movements) and to assess how these two age groups differ regarding the number of safe crossings, virtual crashes, and missed crossing opportunities. The recording of eye and head movements represents the novelty of this approach.

The following hypotheses were tested: Older participants were expected to behave in a less secure manner compared to younger ones, with more virtual crashes, fewer missed crossing opportunities, and more visual fixations on the floor. For both age groups, more missed opportunities were expected at a lower speed of approaching cars, and more virtual crashes at a higher speed, since crossing decisions are based on the vehicle’s distance instead of speed [11, 12].

## Methods

### Participants and ethical approval

20 healthy, younger participants (8 men, aged between 23 and 28 years old, M = 25.15, SD = 1.81) and 20 healthy, older participants (12 men, aged between 65 and 79 years old, M = 70.50, SD = 4.43 years old) were recruited to participate in this study. Older participants were recruited through the “Senior University of Bern”. Younger participants were recruited through advertisement at the University of Bern. Participation in the experiment was free. Inclusion criteria were: a MoCA [23] score > 26 for both age groups, age range between 20 and 30 years old for the younger group, and age range between 60 and 80 years old for the older group. Exclusion criteria were: severely impaired motor abilities (e.g., resulting from chronic pain), inability to stand for about 1 h, or restricted visual field (less than 140° on the horizontal dimension). All subjects had a post-secondary education degree. Due to poor quality of eye and head movements data, only 18 older and 18 younger subjects were included in the analysis.

The study was carried out in accordance with the latest version of the Declaration of Helsinki, Ethical Approval was provided by the Ethics Committee of the Canton of Bern, Switzerland.

### Experimental setup

The experiment was comprised of two blocks of about 30 min each. In the first block, a general screening of the cognitive and visual abilities of the participants, as well as the 10 m Gait Speed Test (GST), were conducted. In the second block, the street crossing simulation, preceded by a practice session, was carried out.

All participants gave written informed consent prior to the experiment. The participants were screened for eventual visual field defects by means of finger perimetry. Monocular far visual acuity was assessed by means of a Landolt-Ring chart at a distance of 5 m from the participants [24]. If needed, participants wore their habitual correction (glasses, contact lenses).

Cognitive abilities were assessed with standardized paper-pencil tests. The Montreal Cognitive Assessment (MoCA) was used to check whether participants fulfilled the respective inclusion criterion. The Trial Making Test (TMT) [25], parts A and B, was used to assess visual attention and executive functions, respectively [26]. The Clock Drawing Test (CDT) [27] was used to assess visuo-constructional abilities and abstract thinking [28].

The GST was administered to measure the walking speed of the participants [29]. In this test, participants were asked to walk three times in a corridor for a marked distance of 12 m, at a self-selected walking speed, as they would do while crossing a street, but without time pressure. The measured distance was only of 10 m, because the first and last meter were excluded from analysis due to the acceleration and deceleration (starting and stopping walking) of the participants. Afterwards, participants were asked to stand in front of the same corridor, to imagine themselves walking straight ahead for the same distance, and to estimate their own walking time for three times [14].

### Street crossing

**Fig. 1:**
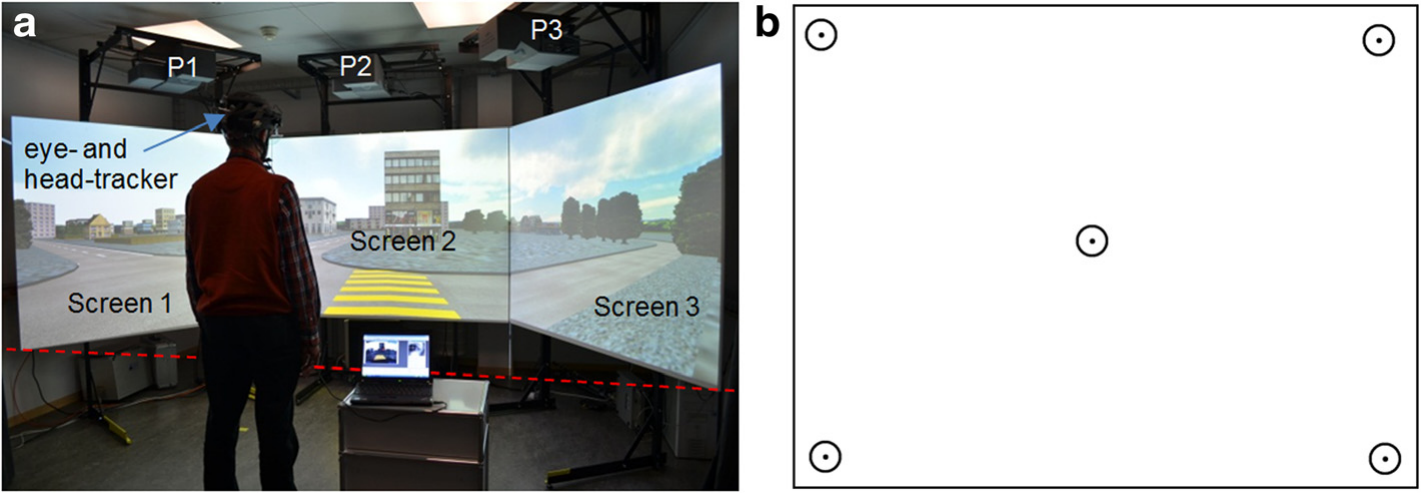
Experimental setup of the street crossing simulation. **a** A participant wearing the eye-tracking system is standing in front of the simulator. Screen 2 is straight in front of the participant. Screen 1 and 3 are tilted by 120° with respect of screen 2, around a vertical line at the edge with it. Images are projected onto the three screens by the projectors P1, P2, and P3. An infrared light beamer, on the right hand of Screen 3, sends light to a detector on the opposite side (red dotted line). When the participant decided to cross the street, he or she was instructed to take a step forward, interrupting the infrared beam between the beamer and the detector. **b** Calibration pattern projected onto screen 2. The central point is at 0° eccentricity. The other four points are at 32°, in the top left, top right, bottom left and bottom right corners

The main experiment of this study was the street crossing simulation. It took place in a temperature-, light-, and noise-controlled room, with a modified version of a driving simulator (F12PI-3/A88, Foerst GmbH, DE). The car component of the driving simulator was removed for the purpose of the experiment. The virtual scene was projected by three projectors (Ultra-Short Focus LCD projector, Sanyo), with a resolution of 1024 × 786 pixels, on three projector screens (1.80 × 1.39 m). The participants were standing in front of them (Fig. 1a). As shown in Fig. 1a, Screen 2 was straight in front of the participants. Screen 1 and 3 were tilted by 120° with respect to screen 2, around a vertical line at the edge with it. This allowed to achieve an immersive environment, resulting in a field of view of 180° on the horizontal plane and 40° on the vertical plane. Three computers running Microsoft Windows 7 operating system (Microsoft, Inc., WA) were used to control the simulation, one for the calculation and the control of the dynamic scenario, and two for the graphics. A photoelectric sensor and an infrared light beamer (Velleman PEM10D, NL) were integrated into the simulator, in order to determine when the participants decided to cross the street, by detecting the interruption of the light beam (Fig. 1a).

In the experiment, the participants were standing in front of a projected cross-walk of a two-way road (Fig. 1a), while cars were driving in the nearer lane from left to right, as it is expected in real life in Switzerland, where the experiment took place. Two scenarios were presented: a slow one, with cars driving at 30 km/h, and a fast one, with cars driving at 50 km/h. In each scenario, six cars were driving at the same, constant speed. Between the third and the fourth car, there was a time gap, and the participants had to choose whether the time gap was long enough to safely cross the street. The time gaps varied between 1 and 7 s, with 1 s increment, as suggested by Dommés and Cavallo [2]. The participants had to indicate their decision to cross the street by taking a step forward, which was recorded by the photoelectric sensor. They were instructed to take into account their own walking speed, as measured by the GST, and to cross only if they could do it safely and without taking any risks, thus, behaving as naturally as possible. 30 trails, with different time gaps and different car speeds, were presented in a randomized order: time gaps of 1 and 7 s were presented once, time gaps of 2 and 6 s were shown twice, and the time gaps of 3, 4, and 5 s were presented three times, making 15 trials, per one speed value.

During the simulation, the participants wore a helmet with a head-mounted eye- and head-tracking system (Fig. 1a). Eye-movements were recorded using a HED 4 (SMI iView X HED, DE) video-based corneal reflection tracker. After performing a five-point calibration on the central screen (points at 0° and 32°, as shown in Fig. 1b), the system tracked the gaze position for the whole duration of the experiment. Additionally, participants’ head movements were recorded by a passive head-tracking system (Atracsys InfiniTrack 500, CH). A passive marker with four reflective spheres, with a diameter of 13 mm each, was fixed on the back of the helmet. A sensor placed behind the participant captured the position of the head in three dimensions for the whole duration of the experiment.

### Data analysis

The outcome measures of the cognitive tests were the performance on the single tasks, and the respective time until completion of the same tasks. For the far visual acuity, only performance was recorded. For the street crossing simulation, the behavioral outcomes were the number of safe crossings, the number of virtual crashes, and the number of missed opportunities. The sum of all the three outcomes was always equal to the total number of repetitions, namely 30. A safe crossing was defined as a decision to cross the street that would not lead to any collision with the oncoming vehicles. A virtual crash was defined as the opposite of the safe crossing, namely a decision to cross the street that would lead to a collision with a vehicle. More in depth, the number of safe crossings and virtual crashes was computed in the following way: Given the moment when the participants decided to cross, the speed of the vehicles and the own walking speed of the participants, measured with the GST, a virtual crash was identified as an event where the vehicle and the pedestrian were at the same point in space, and at the same instant in time. A missed opportunity was defined as a decision to let the vehicle pass even if the time gap was sufficient to safely cross the street.

Eye-tracking outcomes were assessed in terms of number of visual fixations on three regions of interest (ROI): left part of the screen, the floor below the screen and right part of the screen (Fig. 3a). Head-tracking outcome was the number of head movements rightward, leftward, and downward. The gaze behavior was calculated by taking into account both eye and head movements [16]. In a further analysis, head movements alone were also analysed. Eye-tracking accuracy, during the calibration, was calculated as the distance between the position of a detected fixation in a calibration point and the actual position of that calibration point.

The software Statistica (StatSoft, Inc, OK) was used for statistical analyses. Independent sample Mann-Whitney U tests were used to compare performance between the two groups in the general screening tests. To test differences in the actual, as compared to the estimated walking speed, between the two groups, independent sample Mann-Whitney U tests on the difference between the two walking speeds was used. Gender differences in the own walking speed, the estimated walking speed, and the difference between actual and estimated walking speed, were also tested with independent sample Mann-Whitney *U* test. Performance on street crossing simulation was firstly assessed with a Wilcoxon Signed Rank Test on factor Speed (30 and 50 km/h), in the younger and older groups, respectively. Then a Mann-Whitney *U* Test was used to compare the two age groups, for 30 and 50 km/h, respectively. Visual exploration behavior was firstly assessed with a Friedman’s Two-Way Analysis of Variance by Ranks on factor Location (left part of the screen, right part of the screen and space below the screen), in the younger and older group, respectively. Secondly, Wilcoxon Signed Rank Tests were used to compare different locations (left and right parts of the screen, left part of the screen and space below the screen, right part of the screen and space below the screen) within each age group. Then an independent sample Mann-Whitney *U* Test was used to compare the two age groups in the left part of the screen, the right part of the screen and the space below the screen, respectively. Holm-Šidàk corrected p-values were used to take into account multiple comparisons [30]. The following formula was used to compute the corrected p-values:

$$ {p}_{corrected}=1-{\left(1-{p}_{original}\right)}^{C-i+1} $$

where *C* represents the total number of comparisons, and *i* = 1 … *C* represents the iteration index. The significance level for the corrected p-values was set to 0.05.

## Results

### General screening

A summary of the main results of the general screening is shown in Table 1.

**Table 1:** Summary of the results of the general screening

Variable	Younger participants^a^	Older participants^a^	p-value
Age	25.00 (1.78)	70.22 (4.11)	
General screening			
Far visual acuity (M-units)	0.98 (0.26)	0.67 (0.22)	0.001
TMT-A (time in s)	18.87 (4.21)	37.58 (10.28)	<0.001
TMT-A (errors)	0.00 (0.00)	0.06 (0.24)	n. s.
TMT-B (time in s)	43.21 (19.98)	86.20 (30.86)	<0.001
TMT-B (errors)	0.00 (0.00)	1.11 (1.13)	<0.001
CDT	6.94 (0.24)	6.06 (1.21)	0.034
GST (m/s)	1.39 (0.22)	1.34 (0.16)	n. s.
Estimated speed (m/s)	1.44 (0.30)	1.54 (0.26)	n. s.

^a^ Data in the table represent mean values and standard deviations [M (SD)] out of 18 participants per group

The younger and the older groups significantly differed regarding far visual acuity [U(34) = 62.0, *p* = 0.001], with better performance in the younger group. For both the TMT-A and -B, significant group differences were found: the younger group was faster in both tests ([U(34) = 9.0, *p* < 0.001] for TMT-A and [U(34) = 27.0, *p* < 0.001] for TMT-B), and showed better performance only on the TMT-B [U(34) = 63.0, *p* < 0.001]. The younger group showed better performance also on the CDT [U(34) = 95.5, *p* = 0.034]. No significant group differences were found for the GST [U(34) = 144.0, *p* = 0.584], nor the self-estimated walking speed [U(34) = 119.0, *p* = 0.181]. A significant overestimation of their own walking speed compared with the GST was found in the older group [U(34) = 95.0, *p* = 0.034]. No gender differences were found in the walking speed ([U(16) = 28.5, *p* = 0.315] in the younger group, [U(16) = 31.0, *p* = 0.536] in the older one), neither in the estimated walking speed ([U(16) = 37.0, *p* = 0.829] in the younger group, [U(16) = 23.5, *p* = 0.179] in the older one), nor in the difference between actual and estimated walking speed ([U(16) = 38.5, *p* = 0.897] in the younger group, [U(16) = 24.0, *p* = 0.211] in the older one).

### Street crossing simulation

**Fig. 2:**
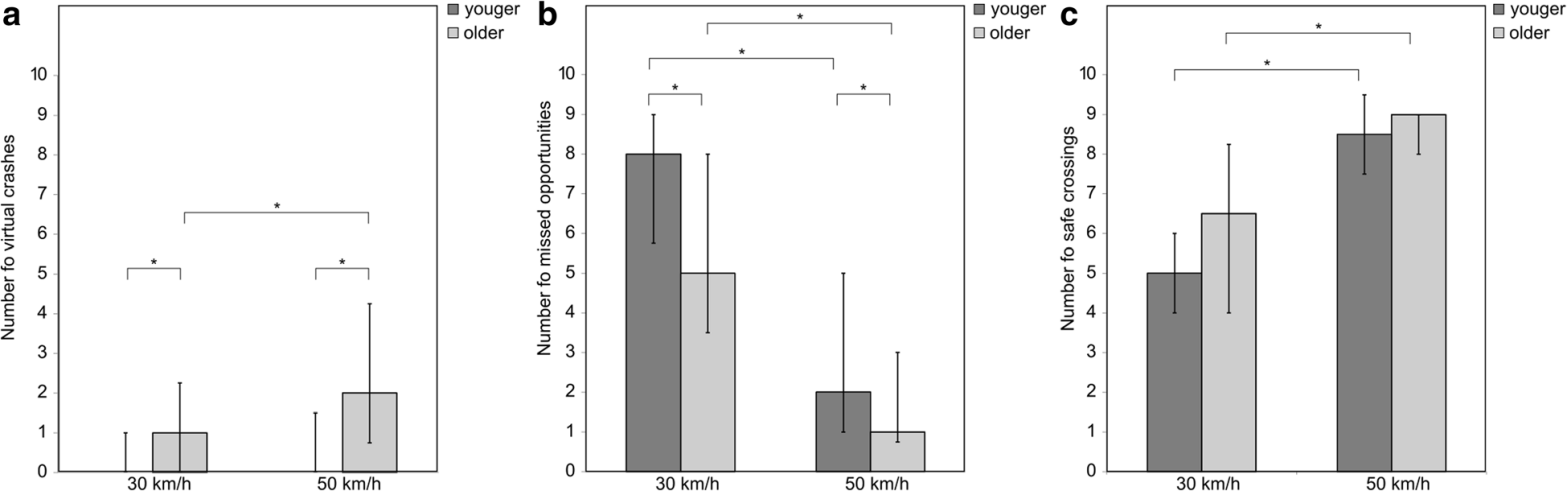
Results of the street crossing simulation. **a** Median number of virtual crashes out of 30 repetitions, at 30 km/h and 50 km/h, for the younger and the older group. **b** Median number of missed opportunities out of 30 repetitions, at 30 km/h and 50 km/h, for the younger and the older group. **c** Median number of safe crossings out of 30 repetitions, at 30 km/h and 50 km/h, for the younger and the older group. Error bars represent the interquartile range. * depicts p_corrected_ < 0.05

The results of the street crossing simulation are shown in Fig. 2.

A significant effect of factor Speed was found for the number of safe crossings in the younger group [Z = −3.57, p_corrected_ < 0.001] and the older one [Z = −3.22, p_corrected_ = 0.001], the number of virtual crashes in the older group only [Z = −3.08, p_corrected_ = 0.002], and the number of missed opportunities in the younger group [Z = −3.75, p_corrected_ < 0.001] and the older one [Z = −3.74, p_corrected_ < 0.001]. A significantly higher number of virtual crashes and safe crossings, and a significantly lower number of missed opportunities, were observed at higher compared to lower speed. A significant effect of factor Age was found for the number of virtual crashes at 30 km/h [U = 90.0, p_corrected_ = 0.043] and at 50 km/h [U = 99.5, *p* = 0.047] and the number of missed opportunities at 30 km/h [U = 89.5, p_corrected_ = 0.041] and at 50 km/h [U = 97.5, p_corrected_ = 0.040], but not for the safe crossings at 30 km/h [U = 110.0, p_corrected_ = 0.989], nor at 50 km/h [U = 156.0, p_corrected_ = 0.864]. The older group showed a significantly higher number of virtual crashes and a significantly lower number of missed opportunities as compared to the younger group, irrespective of the speed condition.

### Eye and head movement analysis

**Fig. 3:**
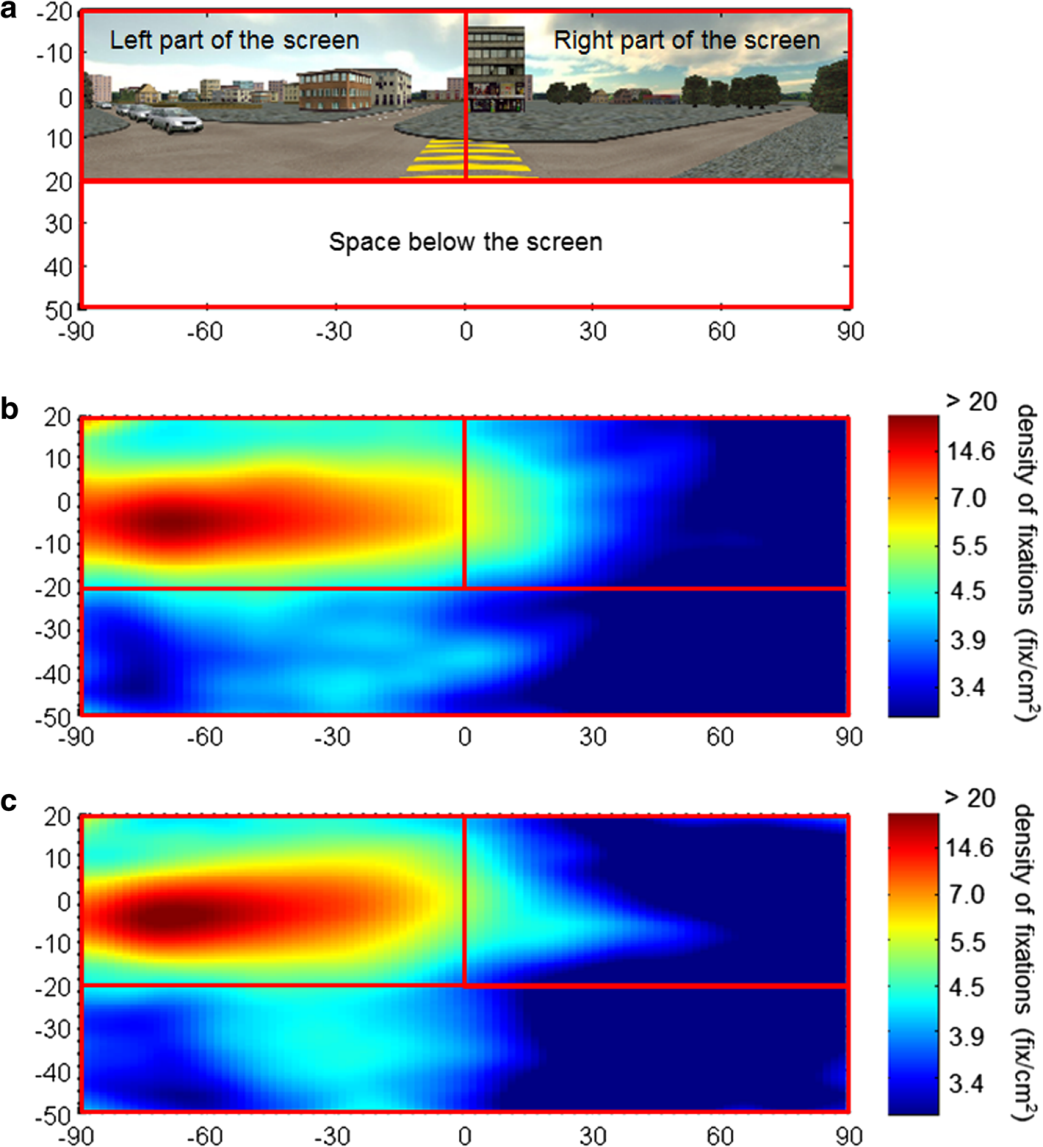
Density of fixations. Density of fixations in the younger and older group for the entire duration of the experiment. **a** Visual scene, as it was presented to the participants. In the present depiction, the images on the three screens have been aligned for better visualization. **b** Density of fixations in the younger group. **c** Density of fixations in the older group

The accuracy of the eye and head movement data was (1.28 ± 0.94)° for the younger group, and (1.52 ± 1.09)° for the older one. The graph in Fig. 3b, c represents the density of fixations on the visual scene in the younger and the older group for the entire duration of the experiment. The three ROIs are marked. This qualitative analysis revealed that the two groups had similar patterns in their visual exploration behavior, with most of the fixations towards the left part of the screen. The point where the vehicles were expected to appear is a hot spot, with more than 20 fixations/cm^2^, but also the area around it was largely explored. The extent of this area reaches the right part of the screen in both groups, but it is larger in the younger participants. The older group explored more often the region below the screen.

**Fig. 4:**
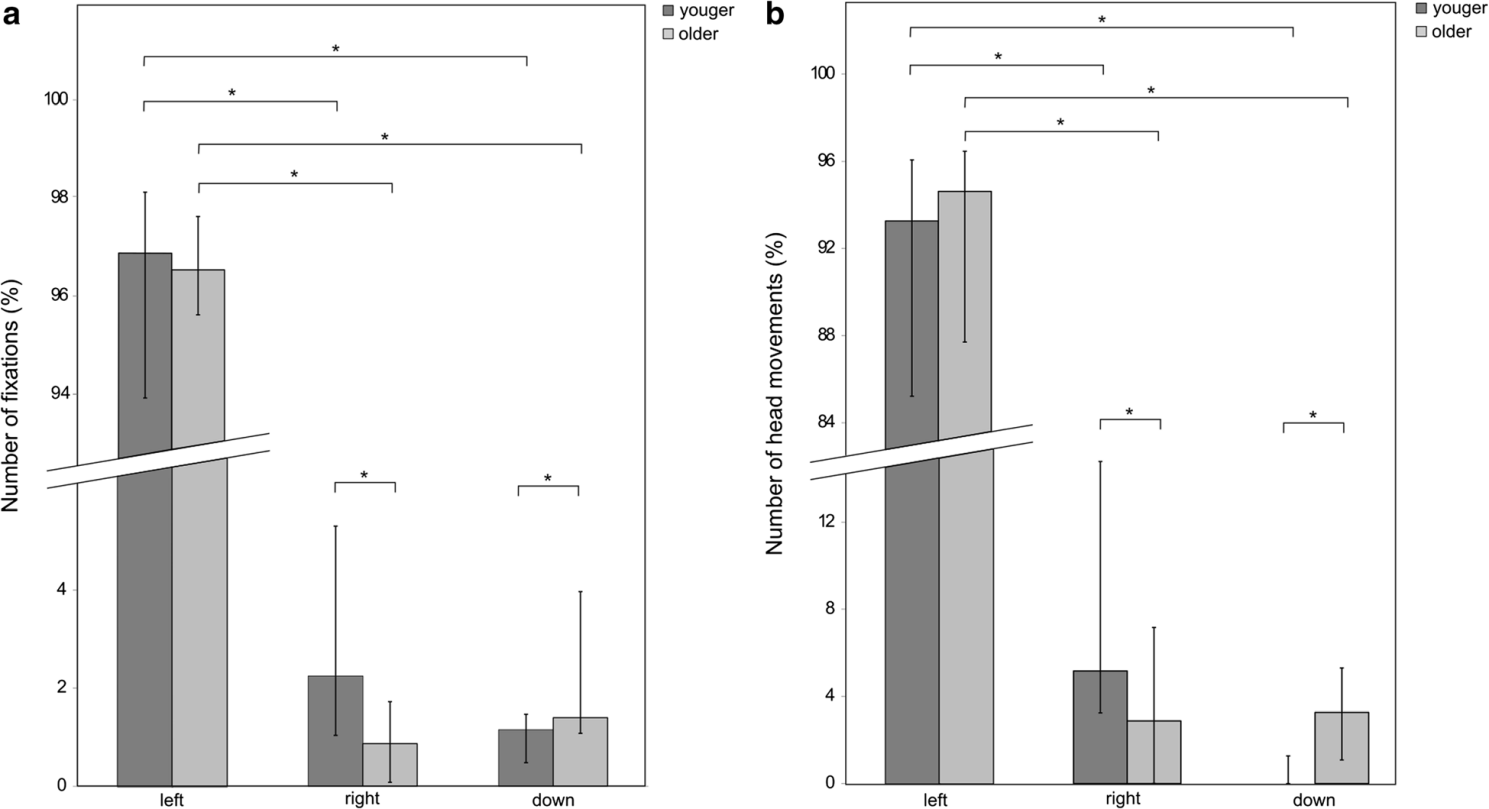
Analysis of the visual exploration behavior. Results of the analysis of the visual exploration behavior taking into account head movements, and of the head movements alone. Bars represent the median, error bars represent the interquartile range. **a** Visual exploration behavior. **b** Head movements. A similar pattern was observed for the gaze behavior and the head movements: Both groups had significantly higher number of fixations and head movements towards the left part of the screen. The older group explored less the right part of the screen, and more below the screen, as compared to the younger group. * depicts p_corrected_ < 0.05

The results of the statistical analysis on the visual exploration behavior and the head movements are shown in Fig. 4.

For the visual exploration behavior, a significant main effect of factor Location (*χ*^2^(2) = 29.78, *p* < 0.001 for the younger group, *χ*^2^(2) = 28.79, *p* < 0.001 for the older one) was found. A similar pattern was found for the number of head movements, with a main effect of factor Location (*χ*^2^(2) = 33.37, *p* < 0.001 for the younger group, *χ*^2^(2) = 27.41, *p* < 0.001 for the older one). Wilcoxon Signed Rank Tests revealed that both groups produced most fixations on the left part of the screen ([Z = −3.72, p_corrected_ < 0.01] and [Z = −3.72, p_corrected_ < 0.01] in the comparison left and right parts of the screen for the younger and the older groups, respectively, [Z = −3.72, p_corrected_ < 0.001] and [Z = −3.72, p_corrected_ < 0.001] in the comparison left part of the screen and space below the screen, for the younger and the older groups, respectively), and also most head movements were directed towards the left part of the screen ([Z = −3.72, p_corrected_ < 0.01] and [Z = −3.72, p_corrected_ < 0.01] in the comparison left and right parts of the screen for the younger and the older groups, respectively, [Z = −3.72, p_corrected_ < 0.001] and [Z = −3.72, p_corrected_ < 0.001] in the comparison left part of the screen and space below the screen, for the younger and the older groups, respectively). Mann-Whitney U Tests on Age showed that, as compared to the older group, the younger group explored more often the right part of the screen [U = 85.0, p_corrected_ = 0.041] and less often below the screen [U = 90.0, p_corrected_ = 0.043]. Head movements were also more frequent towards the right part of the screen [U = 77.5, p_corrected_ = 0.012] and less frequent towards the space below the screen [U = 65.5, p_corrected_ = 0.006] in the younger than in the older group.

## Discussion

The present study was designed to expand the knowledge about the decision making process during street crossing, using eye- and head-tracking technology, and to compare the crossing behavior in younger and older pedestrians.

For the visual exploration behavior, the percentage of fixations towards the left part of the screen was very high for both groups. In a previous study, Hollands et al. [31] found that healthy subjects at a street crossing intersection direct the majority of their fixations onto objects that lied in front of them or onto the oncoming vehicles. We could confirm this pattern because, as shown in Fig. 3b, c, more than 5.5 fixations/cm^2^ were located in the central and left parts of the screen, where the vehicles were expected to appear, with a hot spot of > 20 fixations/cm^2^ on the vehicles. Furthermore, the area with such a high density of fixations is quite large, suggesting that both groups not only looked at the lane with the approaching cars, but also at other elements around it. These findings are in line with the ones by Geruschat et al. [32], who found that subjects often fixate the area where cars are expected to appear but, when no cars are present, their gaze would fall on buildings or trees within the same area. The percentage of fixations below the screen was higher in the older group as compared to the younger one. Previous research, indeed, showed that, with age, people need more time to plan precise stepping movements [33, 34]. This may result in more fixations to the ground and, in turn, less attention to the traffic, with consequent poorer performance on the street crossing task. The number of head movements and visual fixations towards the right part of the screen resulted higher in the younger group, and, as shown in Fig. 3b, concentrated in the central part of the scenario. This could represent a further confirmation of a safety-relevant behavior of the younger group, focusing also on the target to reach, namely the other side of the street, in order to correctly judge the distance to walk.

The results of the general screening showed, as expected, age effects in global visual functions and cognitive testing. Older people showed significantly worse performance in the far visual acuity test, although they wore their habitual correction, when needed. They also showed a higher number of errors in the TMT-B, and obtained lower scores in the CDT. These cognitive differences can be expected with increasing age [35]. Furthermore, older people needed more time to complete the TMT-A and -B, and resulted to be globally slower than younger participants. This factor was shown to be related with executive functions during walking [36], and plays an important role in street crossing, where fast decisions have to be taken [37]. The results of the GST and the estimation of one’s own walking speed showed that older participants significantly overestimated their walking speed, despite the fact that no significant group differences were found neither in the actual walking speed, nor in estimating the own walking speed. This may indicate poor compensation for declining cognitive abilities. Older pedestrians may not be aware of, or deny, their diminished abilities, thus choosing gaps that are too short to accommodate their slower walking speed [9].

The results of the street crossing simulation showed that both age groups performed more safe crossings and less missed opportunities in the condition with faster cars. However, more virtual crashes were found at higher speed. This suggests that decisions of all age groups are based rather on the distance than on the speed of an approaching car. This is reflected in the fact that, for a given time gap, lower speeds are associated with shorter car distances than higher speeds [2]. When vehicles were closer, their angular size was bigger and their visual expansion as they approached was greater. This ‘looming’ effect [38] can lead to a feeling of danger and, therefore, to a rejection of an acceptable gap. In addition, considering the higher number of virtual crashes and fewer missed opportunities in the older group compared to the younger one, it can be argued that, for long distances, older people cross more often regardless of the oncoming vehicle’s speed. This leads to greater risk-taking behavior, and increases the possibility of an accident with a fast-moving car. It is likely that older pedestrians have more difficulties in analyzing the movement of an approaching car when this is still far away and, thus, at the edge of the visual field. These results are even more distinctive under time constraint [17]. In agreement with the results of the cognitive assessment, it can be argued that younger people have more cognitive capacities, and can work more efficiently under time pressure, whereas, under the same time conditions, older people often take risky decisions and make more errors. In addition, the numerous missed opportunities of younger participants, as well as the fewer virtual crashes, can be interpreted as a very safe weighting of unclear situations, i.e., letting the vehicles pass in case of doubt.

## Conclusions

The present study has a few limitations. In real-life street crossing settings, there is an interaction between the pedestrian and the driver of a car, by means of eye contact or gestures. A more interactive virtual reality environment might address this issue, but, like in other studies, this interaction was not implemented in the present study. Another limitation of our study was the use of a traffic scenario with only one way, with cars approaching only from left to right. Nevertheless, in other studies [14], two ways were used, but the time gaps were always synchronized and, thus, did not reflect real conditions with two-way traffic. The noise of the cars changing with their speed might also influence the decision to cross a road, but our setup could not reproduce 3D sound, and auditory stimuli were, thus, not taken into account in the measurement.

Our approach presents several strengths. Street crossing behavior was investigated in a safe environment, where the visual exploration could be measured and analyzed. Eye-tracking technology opened new insights into the visual exploration behavior during everyday life activities, such as street crossing. Furthermore, the simulated environment allowed a standardized comparison across all participants. In our study, the participants were standing in front of three screens covering a large proportion of the visual field, and inducing a realistic impression of the size of the oncoming vehicles. Moreover, the decision to cross was indicated in an active fashion, because a step forward was required, and our participants behaved more likely as in a real-world street crossing setting.

In summary, our study showed differences in street crossing performance between the investigated age groups. Younger participants acted in a safer way, which was reflected in fewer crashes and more missed opportunities. The more risky behavior of older participants could be explained by the complexity of the task. Many variables have to be simultaneously evaluated and, given the reduced cognitive and visual abilities of older people, this might result in difficulties, especially under time pressure. For both age groups, we found a tendency to base decisions to cross a street on the distance of the oncoming cars, rather than on their speed. This pattern can lead to very dangerous decisions when cars are driving fast. Eye- and head-tracking technology, the novel element of the present study, allowed to measure visual exploration behavior and head movements of the participants. We found, indeed, that the older participants looked more often at the ground and, in turn, paid less attention to the traffic.

The practical implications of our study go in the direction of improving safety of seniors: as suggested by Dommes et al., car-free islands in the middle of two-way streets could help the older pedestrians to cross in two stages, introducing a break in the walk, decreasing the time spent in the street, and dividing the cognitive load by factor two [14]. Speed limit management, with the increase in the number of speed ramps, narrower streets, and zones at 30 km/h, would considerably improve senior safety too. Training programs can be used as well as a supplement to safety regulations and pedestrian-friendly infrastructure measures (e.g., mixed physical-cognitive trainings) [2].

## Abbreviations

GST: gait speed test
MoCA: montreal cognitive assessment
TMT: trial making test
CDT: clock drawing test
ROI: region of interest
